# Longitudinal change in health-related quality of life after intensity-modulated radiation monotherapy for clinically localized prostate cancer

**DOI:** 10.1007/s11136-013-0603-6

**Published:** 2013-12-13

**Authors:** Shinya Yamamoto, Yasuhisa Fujii, Hitoshi Masuda, Shinji Urakami, Kazutaka Saito, Takuyo Kozuka, Masahiko Oguchi, Iwao Fukui, Junji Yonese

**Affiliations:** 1 Department of Urology, Cancer Institute Hospital, Japanese Foundation for Cancer Research, 3-8-31, Ariake, Tokyo, Koto-ku 135-8550 Japan; 2 Department of Radiation Oncology, Cancer Institute Hospital, Japanese Foundation for Cancer Research, Tokyo, Japan

**Keywords:** Prostate cancer, Quality of life, Radiotherapy, Intensity-modulated

## Abstract

**Purpose:**

The purpose of the study is to assess longitudinal changes in general and disease-specific health-related quality-of-life (HRQOL) indices after intensity-modulated radiotherapy (IMRT) monotherapy for patients with localized prostate cancer (PCA).

**Methods:**

Between 2006 and 2010, 91 patients with localized PCA underwent IMRT monotherapy and were enrolled into this prospective study. At baseline, and at 3, 6, 12, and 24 months after IMRT, the general and prostate-specific HRQOL were estimated using physical (PCS) and mental component summaries (MCS) calculated using the Medical Outcomes Study 8-Item Short Form Health Survey and Expanded Prostate Cancer Index Composite (EPIC).

**Results:**

For 2 years, there were no significant changes in EPIC scores in all subscales of urinary domain, hormonal function, and bother. Bowel and sexual function scores decreased after IMRT and did not return to those at baseline (*p* = 0.006 and < 0.001, respectively). PCS began to decrease at 3 months after IMRT and then returned to the baseline score at 24 months. In contrast, the MCS score began to significantly increase after IMRT, and thereafter the score remained constant until 24 months (*p* < 0.001). On multivariate logistic regression analysis, urinary (*p* = 0.003) and sexual functions (*p* = 0.0005) at baseline were identified as significant predictors of EPIC urinary irritative/obstructive score and sexual function at 24 months after IMRT.

**Conclusion:**

Urinary function, including irritative/obstruction symptoms and hormonal function, was not affected by IMRT. However, bowel and sexual function decreased after IMRT. These findings will provide important information for PCA patients considering IMRT.

## Introduction

External-beam radiation therapy (EBRT) and radical prostatectomy (RP) have been the gold standard treatments for clinically localized prostate cancer (PCA), and they have resulted in an excellent disease-free survival outcome for patients [1, 2]. Therefore, since EBRT improves patients’ chances of achieving normal longevity, EBRT is believed to yield a good health-related quality of life (HRQOL) with excellent oncological outcomes [1–3].

Intensity-modulated radiotherapy (IMRT), which recently replaced conventional radiation therapy [1, 2], has become a popular treatment for patients with clinically localized PCA. A higher dose of radiation can be delivered to the prostate and seminal vesicles during IMRT, while lower amounts of radiation reach the adjacent organs, such as the rectum, urinary bladder, and urethra [4, 5]. Alicikus et al. [3] reported that the 10-year likelihood of developing grade 2 and grade 3 late genitourinary toxicity in patients treated with IMRT was 11 and 5 %, respectively, and the 10-year actuarial incidence of developing post-IMRT erectile dysfunction was 44 %. Several investigators have also reported a longitudinal change in HRQOL after IMRT [6–11]. However, because the patients in most of these reports underwent IMRT combined with some androgen deprivation therapy (ADT) [6, 7, 9, 10], HRQOL after IMRT alone has not been well studied. Moreover, because a researcher has analyzed the change in HRQOL after IMRT using the University of California-Los Angeles Prostate Cancer Index (UCLA-PCI) [6], the effect on irritative symptoms and hormonal function after IMRT also remains unclear. The Expanded Prostate Cancer Index Composite (EPIC) was developed in 2000 to include questions concerning irritative/obstructive symptoms and hormonal functions in the UCLA-PCI [12]. Recently, there have been a few large studies using the EPIC for patients who underwent EBRT alone [13, 14]; however, some patients received three-dimensional conformal radiotherapy. These reports were not longitudinal studies. Before treatment, patients are naturally concerned with possible changes in various functions (for example, urinary and bowel functions) following treatment. For this reason, it is very important for patients who are considering IMRT monotherapy to have information about the longitudinal changes in QOL after treatment. Thus, we prospectively evaluated the longitudinal changes in general and disease-specific HRQOL after IMRT monotherapy for patients with localized PCA using the EPIC and the Medical Outcomes Study 8-Item Short Form Health Survey (SF-8).

## Patients and methods

Between August 2006 and February 2010, 96 Japanese patients, who were diagnosed with clinically localized PCA, underwent IMRT at our hospital and enrolled in this prospective HRQOL study. All patients had a histologic diagnosis of PCA from needle biopsy specimens. Clinical staging was determined using digital rectal examination, abdominopelvic computed tomography, pelvic magnetic resonance imaging, and bone scans. After the patients were informed of their cancer diagnosis and staging, we proposed RP and IMRT for localized PCA patients. The final decision on treatment strategy was made by the patients themselves. Of the 96 patients, 91 patients with pretreatment HRQOL data and data from at least three later times were finally included in this study. No patients received any ADT during this study period. This study was approved by the Institutional Review Board of our hospital.

Planning CT scans were obtained from patients with a full bladder and an empty rectum. The clinical target volume (CTV) was defined as the base of the seminal vesicle and the prostate with a 5-mm margin excluding the rectum. The margins for defining the planning target volume (PTV) were 5 mm in all directions of the CTV. Daily positioning verification was performed before every treatment. Electronic portal images of two orthogonal pelvic views were taken with a full bladder and no rectum preparation, and they were compared with the digitally reconstructed radiographs from the planning CT scan, using bone landmarks.

After informed consent was obtained, the general and prostate-specific HRQOL were estimated using SF-8, EPIC (50-item), and the International Prostate Symptom Score (IPSS). These questionnaires had already been translated into Japanese, and their validity and reliability have been tested previously [15–17]. The SF-8 contains 8 items: general health, physical functioning, physical role, bodily pain, vitality, social functioning, mental health, and emotional role [15]. In the present study, physical (PCS) and mental component summaries (MCS), which were constructed from the 8 items, were used to evaluate the general HRQOL [15]. PCS and MCS were measured using the norm-based scoring method, which is based on a large-scale population study conducted in Japan. EPIC scores were divided into four domains (urinary, bowel, sexual, and hormonal), and for each domain, the summary score and two subscale scores (function and bother) were constructed [16]. The urinary domain also includes urinary incontinence and urinary irritative/obstructive subscales [16]. Each subscale score was calculated on a scale of 0–100 with higher scores representing better quality of life (QOL). The IPSS contains 7 items about the frequency of irritative and obstructive urinary symptoms [17]. Each question is answered on a scale of 1–5. Scores were added together, with a possible total ranging from 0 to 35, with higher scores representing worse QOL. The questionnaires were prospectively administered at five time points: before IMRT (baseline), and at 3, 6, 12, and 24 months after IMRT. The questionnaire was personally administered at each follow-up visit or mailed at regular intervals to the patient with a prepaid envelope to assist with returning the questionnaire. The patients voluntarily returned the self-reported questionnaire by mail, at the next follow-up visit or in the waiting room. The submission rates for the questionnaire were 91 (100 %), 87 (96 %), 89 (98 %), 90 (99 %), and 85 (93 %) at baseline, 3, 6, 12, and 24 months after IMRT, respectively.

All patients were also divided into two groups by pre-IMRT IPSS score as follows: the good urinary function group (<8 points) and the poor urinary function group (8 or more points) [18, 19]. The longitudinal changes in the four urinary subscales in the EPIC were evaluated in each group.

Erectile function at baseline was analyzed using the response to EPIC question 18 (“How would you describe the usual quality of your erections during the last 4 weeks?”). Patients selecting 1 or 2 (1, none at all; 2, not firm enough for any sexual activity) were classified as impotent, with all other responses defined as potent. The longitudinal changes in sexual function and bother in the patients who were potent at baseline were also analyzed.

All scores are presented as mean ± standard deviation (SD). Changes in function and bother scores throughout the 2-year follow-up after IMRT were tested using a mixed-effects linear regression model. Differences in the four urinary subscale scores at baseline between the good and poor urinary function groups were tested using the Mann–Whitney *U* test. An EPIC score decrease greater than one-half of the SD of the baseline value for each domain was considered a clinically significant decrement [20]. Multivariate logistic regression analysis was used to assess predictors of urinary irritative/obstructive symptoms, incontinence, bowel function, sexual function, PCS, and MCS at 24 months after IMRT. Age, body mass index (BMI), comorbidity status, the use of phosphodiesterase-5-inhibitor (PDE-5-I), total IPSS score at baseline, incontinence score at baseline, and sexual function score at baseline were evaluated as possible predictors. All *p* values were two-sided. A *p* value less than 0.05 was considered statistically significant. Statistical analyses were performed using JMP version 9 (SAS Institute Inc., Cary, NC, USA).

## Results

### Patient population

Table 1 shows the baseline characteristics of the 91 patients. The median age of the entire cohort was 70 years, and the median pre-IMRT PSA level was 7.7 ng/mL. Most of the patients were classified in the low- and intermediate-risk groups according to National Comprehensive Cancer Network guidelines [21]. Prostate-specific antigen (PSA) failure was defined according to the Phoenix consensus definition of the PSA nadir plus 2 ng/mL. No patients had PSA failure during this study period. The IMRT dose administered to the PTV was 70 Gy in 18 patients (20 %), 72 Gy in 13 patients (14 %), 76 Gy in 1 patient (1 %), and 78 Gy in 59 patients (65 %). The IMRT dose 70 or 72 Gy was administered to all patients until June 2007; thereafter, 78 Gy was administered to the majority of patients.

**Table 1:** Baseline characteristics according to pre-IMRT urinary and sexual functions

Variable	Pre-IMRT urinary function			Pre-IMRT sexual function		
	Good (*n* = 50)	Poor (*n* = 40)	*p*	Potent (*n* = 27)	Impotent (*n* = 64)	^*p*^
Median age (range)	69 (56–78)	70 (58–79)	0.32	66 (57–76)	70 (56–79)	0.008
Median PSA (range) (ng/mL)	7.6 (4.1–28.1)	7.7 (4.1–19.5)	0.78	7.7 (4.0–25.6)	7.6 (4.0–28.1)	0.65
Clinical *T* stage *n*, (%)			0.62			0.06
T1c-2	49 (98.0)	39 (97.5)		25 (92.6)	64 (100.0)	
T3a	1 (2.0)	1 (2.5)		2 (7.4)	0 (0.0)	
Biopsy GS *n*, (%)			0.76			0.25
4–6	8 (16.0)	6 (15.0)		5 (18.5)	9 (14.1)	
7	39 (78.0)	30 (75.0)		19 (70.4)	50 (78.1)	
8–10	3 (6.0)	4 (10.0)		3 (11.1)	5 (7.8)	
Median BMI (range) (kg/m^2^)	23.7 (19.1–27.5)	22.8 (15.5–35.3)	0.73	22.3 (19.1–25.9)	23.5 (15.5–35.3)	0.30
Having smoking history *n*, (%)	24 (53.3)	19 (47.5)	0.59	16 (64.0)	27 (44.3)	0.09
Comorbidity *n*, (%)						
HT	20 (40.0)	13 (32.5)	0.46	7 (25.9)	27 (42.2)	0.14
DM	10 (20.0)	6 (15.0)	0.53	2 (7.4)	14 (21.9)	0.08
CVD	4 (8.0)	1 (2.5)	0.24	1 (3.7)	4 (6.3)	0.61
Median dose (Gy)	78 (70–78)	78 (70–78)	0.32	78 (70–78)	78 (70–78)	0.57
Use of PDE-5-I *n*, (%)	5 (10.0)	7 (17.5)	0.30	7 (25.9)	5 (8.5)	0.03

*IMRT* intensity-modulated radiotherapy, *PSA* prostate-specific antigen, *GS* Gleason score, *BMI* body mass index, *HT* hypertension, *DM* diabetes mellitus, *CVD* cerebral vascular disease, *PDE*-*5*-*I* phosphodiesterase-5-inhibitor

### Longitudinal changes in HRQOL after IMRT (Fig. 1; Table 2)

**Fig. 1:**
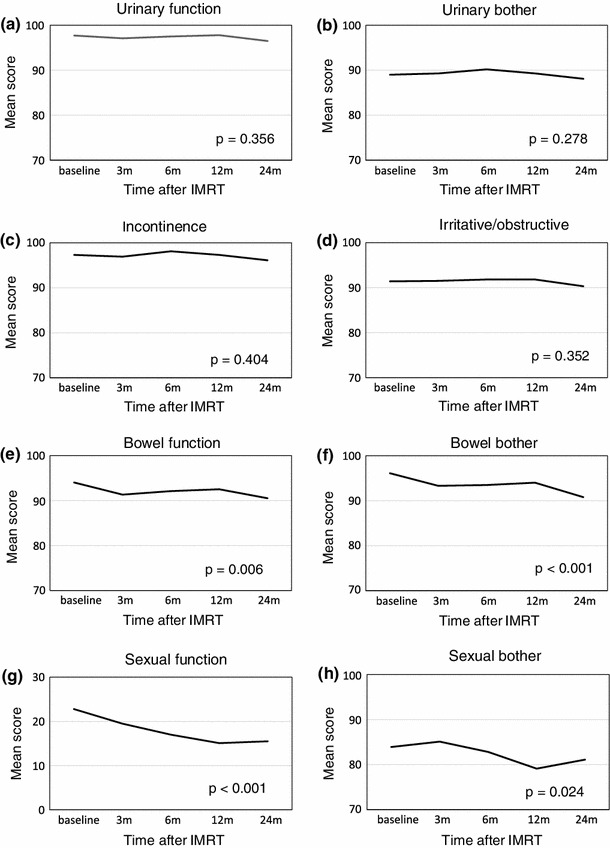

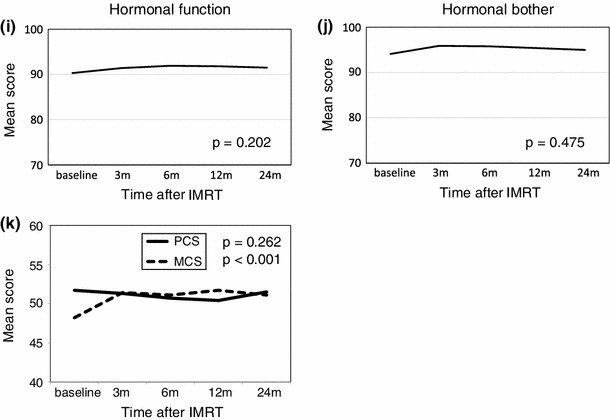
Longitudinal changes in domain and subdomain scores of the EPIC and scores of SF-8 (PCS and MCS) in patients treated with IMRT. High scores indicate better outcomes. **a** Urinary function, **b** urinary bother, **c** incontinence, **d** irritative/obstructive, **e** bowel function, **f** bowel bother, **g** sexual function, **h** sexual bother, **i** hormonal function, **j** hormonal bother, **k** PCS, and MCS

Figure 1 and Table 2 show the EPIC scores for the urinary, bowel, sexual, and hormonal domains at baseline, 3, 6, 12, and 24 months after IMRT in the 91 patients. There was no significant change of the EPIC scores throughout the 2-year follow-up after IMRT in all four urinary subscales. Bowel function and bother EPIC scores began to decrease at 3 months after IMRT, and these scores did not return to baseline for 24 months (bowel function *p* = 0.006; bowel bother *p* < 0.001). The EPIC scores for sexual function gradually decreased after IMRT and then reached a plateau at 24 months; however, the scores at 24 months did not return to those at baseline (*p* < 0.001). Sexual bother scores began to decrease after 3 months, and the scores also did not return to those of baseline for 24 months (*p* = 0.024). The hormonal function EPIC scores did not change throughout the 2-year follow-up after IMRT, and although the hormonal bother scores tended to increase at 3 months after IMRT, there was no significant change throughout the 2 years after IMRT.

**Table 2:** EPIC subdomain scores of patients treated with IMRT monotherapy

Items	Mean score ± SD	Items	Mean score ± SD
Urinary function		Bowel bother	
Baseline (M)	97.7 ± 5.9	Baseline (M)	96.1 ± 6.4
3	97.1 ± 6.8	3	93.3 ± 8.3
6	97.5 ± 6.6	6	93.5 ± 8.1
12	97.8 ± 5.0	12	94.0 ± 7.8
24	96.5 ± 7.6	24	90.8 ± 13.7
Urinary bother		Sexual function	
Baseline (M)	89.0 ± 11.0	Baseline (M)	22.8 ± 20.5
3	89.3 ± 12.0	3	19.5 ± 19.6
6	90.2 ± 10.6	6	17.0 ± 17.6
12	89.3 ± 11.8	12	15.1 ± 16.6
24	88.1 ± 11.6	24	15.5 ± 16.9
Incontinence		Sexual bother	
Baseline (M)	97.3 ± 7.4	Baseline (M)	83.9 ± 22.2
3	96.9 ± 8.7	3	85.1 ± 21.3
6	98.1 ± 7.3	6	82.8 ± 23.6
12	97.3 ± 7.7	12	79.1 ± 26.3
24	96.1 ± 11.1	24	81.1 ± 24.6
Irritative/obstructive		Hormonal function	
Baseline (M)	91.4 ± 9.1	Baseline (M)	90.3 ± 13.6
3	91.5 ± 9.6	3	91.4 ± 10.9
6	91.8 ± 9.0	6	91.9 ± 11.5
12	91.8 ± 9.0	12	91.8 ± 10.5
24	90.3 ± 10.4	24	91.5 ± 12.2
Bowel function		Hormonal bother	
Baseline (M)	94.0 ± 5.9	Baseline (M)	94.1 ± 9.2
3	91.3 ± 9.1	3	95.9 ± 5.9
6	92.1 ± 7.4	6	95.8 ± 8.1
12	92.5 ± 7.8	12	95.4 ± 6.8
24	90.5 ± 10.2	24	95.0 ± 9.3

*EPIC* Expanded Prostate Cancer Index Composite, *IMRT* intensity-modulated radiation therapy, *SD* standard deviation, *M* month

PCS scores began to decrease at 3 months after IMRT, followed by a decrease to the lowest scores at 12 months, and a return to the baseline scores at 24 months. However, the MCS scores began to significantly increase at 3 months after IMRT and they then reached a plateau until 24 months (*p* < 0.001).

Even when all patients were divided into two groups by IMRT dose (low-dose group [less than 76 Gy] and high-dose group [78 Gy]), there was no difference in the longitudinal changes in general and disease-specific HRQOL after IMRT between the two groups (data not shown).

### Longitudinal changes in urinary subscale scores after IMRT for patients stratified by pre-IMRT IPSS scores (Fig. 2)

**Fig. 2:**
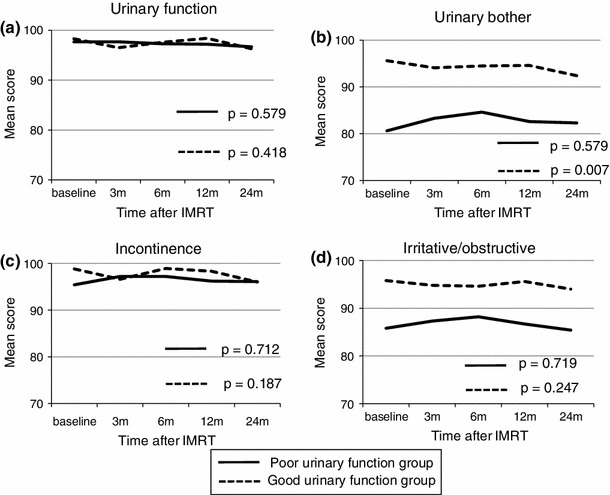
Longitudinal changes in urinary subscales of the EPIC according to pretreatment urinary function. High scores indicate better functional outcomes. Baseline means pretreatment state. Good and poor urinary functions are defined as < 8 points and 8 or more points of pretreatment IPSS score, respectively. **a** urinary function, **b** urinary bother, **c** incontinence, and **d** irritative/obstructive

Before IMRT, 50 (55.6 %) patients had good urinary function and 40 (44.4 %) patients had poor urinary function. There were no significant differences in baseline variables between the two groups (Table 1). At baseline, patients with good urinary function had better urinary bother, incontinence, and irritative/obstructive scores than patients with poor urinary function (urinary bother *p* < 0.001, urinary incontinence *p* = 0.03, and urinary irritative/obstructive *p* < 0.001). In patients in the poor urinary function group, all 4 urinary subscale scores at 3 months after IMRT exceeded those at baseline. In this group, urinary bother, incontinence, and irritative/obstructive scores reached a maximum point 6 months after IMRT, and thereafter, these scores decreased slowly until 24 months (Fig. 2) This trend was particularly notable for the urinary bother score. There was no significant change of the EPIC scores throughout the 2-year follow-up after IMRT in all 4 urinary subscales in the poor urinary function group.

In the group with good urinary function, although there were no significant changes, all 4 urinary subscale scores at 3 months after IMRT were lower than those at baseline. These scores remained constant until 12 months after IMRT and decreased again at 24 months after IMRT, and they did not return to those at baseline, particularly the urinary bother score (*p* = 0.007).

### Sexual function and bother scores after IMRT for potent patients at baseline (Fig. 3)

**Fig. 3:**
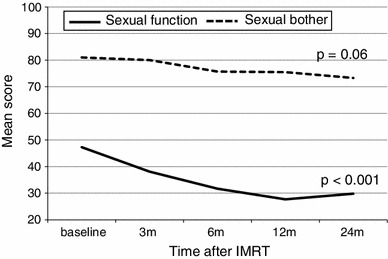
Longitudinal changes in sexual function and bother scores of the EPIC in patients with potency at baseline. High scores indicate better outcomes (sexual function: *solid line*; sexual bother: *dotted line*)

Of the 91 patients, 27 (30 %) were potent before IMRT. The age of the potent patients was younger than that of the impotent patients (*p* = 0.008) and the use of PDE-5-I was more common in the potent patients (*p* = 0.03) (Table 1). The mean sexual function score at baseline was 47.3. As shown in Fig. 3, this score began to decrease at 3 months after IMRT and then stabilized until 24 months (*p* < 0.001). Although the sexual bother score slowly decreased throughout the 2 years after IMRT, there was not a significant change.

### Predictors of QOL (Table 3)

Table 3 shows the multivariate logistic regression analysis of the association between the variables and QOL score decrement at 24 months after IMRT. BMI (*p* = 0.02) and total IPSS score at baseline (*p* = 0.003) were identified as significant predictors of a decrease in EPIC urinary irritative/obstructive scores. Incontinence at baseline (*p* = 0.008) and having smoking history (*p* = 0.03) were identified as significant predictors of a decrease in EPIC incontinence scores. Sexual function at baseline (*p* = 0.0005) was associated with a decrease in sexual function, and age (*p* = 0.02) was associated with a decrease in PCS. No predictor of bowel function and MCS was identified.

**Table 3:** Multivariate analysis of predictors associated with urinary irritative/obstructive, incontinence, bowel, sexual, physical, and psychological functions at 24 months after IMRT

Variable	Irritative/obstructive		Incontinence		Bowel function		Sexual function		MCS		PCS	
	OR (95 % CI)	*p*	OR (95 % CI)	*p*	OR (95 % CI)	*p*	OR (95 % CI)	*p*	OR (95 % CI)	*p*	OR (95 % CI)	*p*
Age	1.01 (0.91–1.13)	0.78	1.10 (0.94–1.35)	0.22	1.08 (1.00–1.19)	0.07	1.11 (0.99–1.26)	0.10	1.10 (0.96–1.30)	0.23	1.13 (1.02–1.29)	0.02
HT (yes vs. no)	1.15 (0.33–3.97)	0.82	2.01 (0.28–15.4)	0.47	0.45 (0.14–1.38)	0.18	0.24 (0.05–0.93)	0.05	1.21 (0.21–6.18)	0.82	0.94 (0.27–3.10)	0.92
DM (yes vs. no)	2.41 (0.50–11.4)	0.26	4.78 (0.50–175.8)	0.22	1.27 (0.32–4.92)	0.73	2.58 (0.54–14.0)	0.24	0.54 (0.03–3.88)	0.60	0.91 (0.18–3.66)	0.90
CVD (yes vs. no)	1.91 (0.08–21.7)	0.62	9.96 (0.38–224.1)	0.15	3.02 (0.24–37.7)	0.37	4.82 (0.35–124.9)	0.25	NA	1.00	1.38 (0.06–13.5)	0.80
Having smoking history (yes vs. no)	1.29 (0.40–4.27)	0.67	8.86 (1.15–169.1)	0.03	1.63 (0.57–4.92)	0.37	2.41 (0.64–10.6)	0.21	0.59 (0.11–2.76)	0.51	0.99 (0.31–3.16)	0.98
									0.86 (0.60–1.15)	0.34	1.15 (0.94–1.42)	0.18
BMI (kg/m^2^)	1.32 (1.06–1.73)	0.02	1.05 (0.74–1.50)	0.77	0.97 (0.80–1.18)	0.78	1.02 (0.82–1.31)	0.83	–	–	–	–
Use of PDE-5-I (yes vs. no)	–	–	–	–	–	–	2.89 (0.54–18.1)	0.23	–	–	–	–
Pre-sexual function (yes vs. no)	–	–	–	–	–	–	0.92 (0.88–0.96)	0.0005	–	–	–	–
Pre-IPSS (≤8 vs. ≥7)	8.48 (2.33–41.1)	0.003	–	–	0.94 (0.31–2.78)	0.91	1.56 (0.45–5.65)	0.49	–	–	–	–
Pre-UIR score at baseline	–	–	0.85 (0.73–0.94)	0.008	–	–	–	–	–	–	–	–

*MCS* mental component summary, *PCS* physical component summary, *OR* odds ratio, *CI* confidence interval, *HT* hypertension, *DM* diabetes mellitus, *CVD* cerebral vascular disease, *NA* not appreciable, *BMI* body mass index, *PDE*-*5*-*I* phosphodiesterase-5-inhibitor, *IPSS* International Prostate Symptom Score, *UIR* urinary incontinence

## Discussion

To the best of our knowledge, this is the first time that longitudinal changes in HRQOL after IMRT monotherapy have been evaluated in patients with clinically localized PCA using EPIC, which measures urinary irritative symptoms and hormonal function as well as urinary incontinence and bowel function.

We found that overall urinary function, including urinary irritative/obstructive symptoms, did not change within the 2 years after IMRT. Recently, Goineau et al. [2] reported that urinary symptoms worsened at 2 months after IMRT, but then improved with time in a QOL study after high-dose IMRT. Since our study questionnaire was not administered within 2 months after IMRT, the speculation that impaired urinary function present at 2 months improves by 3 months cannot be conclusively denied. Namiki et al. [6] reported that the urinary function and bother scores were similar after IMRT and conformal radiation therapy (CRT) and did not change for 5 years after treatment. However, the urinary irritative and obstructive symptoms, which are considered to be the main urinary complications of EBRT, were not evaluated because they used the UCLA-PCA, which contains only questions about urinary incontinence. Quon et al. [10] also reported that all four EPIC urinary subscale scores in the patients treated with hypofractionated IMRT did not change in the 2 years after treatment. However, they studied a cohort of patients who underwent ADT for 2–3 years, and the reduction in prostate size due to ADT might have contributed to their urinary function. Therefore, it might be difficult to evaluate true urinary function after EBRT precisely in patients who undergo ADT plus EBRT. In our study, although the changes did not reach significance, urinary symptoms after IMRT tended to improve in patients who had poor urinary function before IMRT. In contrast, patients with good urinary function before IMRT tended to have worse urinary function after IMRT, in particular urinary bother. The reason for this is unclear. This outcome differs from that of patients undergoing brachytherapy, in which transient irritative and obstructive urinary symptoms usually occur for 3–6 months after treatment [22]. Our finding that urinary function after IMRT is affected by pre-IMRT voiding status is significant for patients who wish to undergo IMRT. Improvement of urinary status after surgery in patients with poor urinary symptoms is one of the important advantages of RP [23]. Thus, IMRT may be suitable for patients with poor urinary symptoms before treatment.

Several investigators have reported that bowel function and bother are worse after EBRT than at baseline [13, 24]. Namiki et al. [6] reported that at 5 years after treatment, bowel function and bother in the patients treated with CRT were significantly worse than those at baseline. However, there were no significant differences between the baseline scores and any of the post-radiation scores at any of the time periods in the patients treated with IMRT. In contrast, bowel function and bother scores after IMRT were lower than those at baseline in our cohort and in the cohort studied by Brassell et al. [11]. Although the reason for the discrepancy between these two studies and the results reported by Namiki et al. is unclear, we propose that there might be slight differences in radiation exposure to the rectum among the three studies in question.

Sexual function scores after IMRT gradually decreased compared to baseline, but had stabilized by 2 years after IMRT. Brassell et al. [11] also recently reported QOL changes at 2 years after IMRT monotherapy using EPIC and concluded that sexual function at 2 years after IMRT monotherapy was slightly decreased. In contrast, Namiki et al. [6] reported that, despite the inclusion of some patients in the cohort who received ADT combined with IMRT, sexual function scores did not change for 5 years after treatment. The reason for this controversial finding is unclear, but the percentage of patients with pretreatment potency in each cohort may have differed.

In our study, sexual bother after IMRT of the patients with potency at baseline did not significantly change for 2 years after IMRT. This result may be explained by the theory that since most Japanese originally have low sexual function [25], they are not concerned about sexual function even if their sexual function decreases further after IMRT. Namiki et al. also investigated sexual function and bother before localized PCA treatment in Japanese and Americans, and concluded that Japanese had poorer overall ability to function sexually than Americans, but there was no difference in sexual bother between Japanese and Americans [26].

In previous reports that used the EPIC [9, 10], evaluation of hormonal function and bother have been greatly affected by ADT because of EBRT and ADT combination treatment. In addition, hormonal function and bother have not been evaluated in the previous reports using UCLA-PCI and EORTC QLQ-PR25 [6, 7, 9]. Accordingly, we believe that our hormonal findings will be very useful for patients who are treated with IMRT alone. Brassell et al. [11] showed that hormonal bother was worse 2 years after treatments in patients with lower incomes and indicated that the effect of economic stress on patients with PCA is one of a few reasons for poorer hormonal bother after treatment in these patients. In contrast, hormonal bother tended to increase after IMRT in this study. The effect of economic stress on hormonal bother might be lower in Japanese patients because the National Universal Health Insurance System covers IMRT for PCA in Japan. In Japan, low-income patients <70 years old can receive IMRT for about 600 US dollars, while those more than 70 years old can receive it for less than 150 US dollars.

To date, there have been few reports on PCS and MCS scores in PCA patients who underwent EBRT. Sugimoto et al. [27] reported that there were no significant differences relative to time after EBRT. They reported that the PCS score, which was decreased in the early period after EBRT, returned to the baseline score at 24 months. In contrast, the MCS score, which was increased in the early period after EBRT, remained stable until 24 months. Despite the differences between EBRT and IMRT, this tendency was the same as our study findings.

On multivariate analysis, pre-IMRT urinary and sexual functions were identified as significant predictors of urinary irritative/obstructive, incontinence, and sexual functions at 24 months after IMRT, respectively. Morton et al. [28] also previously reported the same results as ours. Furthermore, Hashine et al. [22] reported that low pretreatment IPSS scores significantly predicted urinary irritative/obstructive function at 3 years after RP or brachytherapy. Therefore, pretreatment urinary function may be significant to predict urinary function after treatment, regardless of the treatment method.

This study has some limitations. First, the cohort was relatively small and the follow-up period relatively short. It is well known that radiation therapy has late toxicities [29], so a longer follow-up period is required. Second, the dose of radiation may not have been sufficient to eradicate PCA, because 34 % of the patients received 72 Gy or less. Third, it is unclear whether the definition of potency was suitable, because it was not clearly specified. Fourth, the hormonal function domain of the EPIC has usually been used for patients treated with ADT; however, it has also been used for patients who did not receive ADT in previous reports [13, 30]. Despite these limitations, there have been few previous reports on the longitudinal changes in general and disease-specific HRQOL after IMRT monotherapy for patients with localized PCA, and the results of this study should be useful for patients deciding on treatment strategies. In particular, we should inform patients that urinary function after IMRT is not markedly poor, and that bowel and sexual functions that deteriorate after IMRT do not return to those at baseline. Future studies should include more patients evaluated for a longer period.

## Conclusions

For 2 years, we prospectively evaluated the longitudinal changes in general and disease-specific HRQOL after IMRT monotherapy with the EPIC, SF-8, and IPSS in patients with localized PCA. Urinary functions, including irritative/obstruction symptoms, and hormonal functions were not affected by IMRT monotherapy, but bowel and sexual functioning decreased after IMRT.

It is important for patients to evaluate the impact that treatment will have on their quality of life and to make an informed choice based on their pretreatment function when they consider undergoing IMRT monotherapy.

## Abbreviations

EBRT: External-beam radiation therapy
RP: Radical prostatectomy
PCA: Prostate cancer
HRQOL: Health-related quality of life
IMRT: Intensity-modulated radiotherapy
ADT: Androgen deprivation therapy
UCLA-PCI: University of California-Los Angeles Prostate Cancer Index
EPIC: Expanded Prostate Cancer Index Composite
SF-8: Medical Outcomes Study 8-Item Short Form Health Survey
CTV: Clinical target volume
PTV: Planning target volume
IPSS: International prostate symptom score
PCS: Physical component summary
MCS: Mental component summary
SD: Standard deviation
PDE-5-I: Phosphodiesterase-5-inhibitor
